# iTRAQ-based comparative proteomic analysis in different developmental stages of *Echinococcus granulosus*

**DOI:** 10.1051/parasite/2021012

**Published:** 2021-03-05

**Authors:** Xin Li, Song Jiang, Xuhai Wang, Wenqiao Hui, Bin Jia

**Affiliations:** 1 College of Life Sciences, Shihezi University Road Beisi Shihezi 832003 Xinjiang PR China; 2 College of Animal Science and Technology, Shihezi University Road Beisi Shihezi 832003 Xinjiang PR China; 3 Anhui Province Key Laboratory of Livestock and Poultry Product Safety Engineering, Institute of Animal Husbandry and Veterinary Medicine, Anhui Academy of Agriculture Sciences Road Nongkenan Hefei 230031 Anhui PR China

**Keywords:** *E. granulosus*, Differential expression proteins, iTRAQ, Candidate targets for diagnosis or therapeutics

## Abstract

Cystic echinococcosis, caused by infection with the larval stage of the cestode *Echinococcus granulosus*, is a chronic zoonosis. The lifecycle of the *E. granulosus* parasite includes three consecutive stages that require specific gene regulation or protein expression to survive environmental shifts between definitive hosts and intermediate hosts. The aim of the present study is to screen and analyze the stage differential antigens to be considered for vaccine development against *E. granulosus*. By using the iTRAQ (isobaric tags for relative and absolute quantification) method, the differentially expressed proteins were selected from the three consecutive developmental stages of *E. granulosus*: oncosphere, adult tapeworms, and protoscolex. Through a bioinformatics analysis including Clusters of Orthologous Groups (COG), Gene Ontology (GO), and pathway metabolic annotation, we identified some proteins of interest from each stage. The results showed that a large number of differentially expressed proteins (375: oncosphere vs. adult, 346: oncosphere vs. protoscolex, and 391: adult vs. protoscolex) were identified from the three main lifecycle stages. Analysis of the differential protein pathways showed that these differential proteins are mainly enriched in metabolic pathways, Huntington’s diseases, Alzheimer’s diseases, and ribosome metabolic pathways. Interestingly, among these differential proteins, expression levels of paramyosin, HSP60, HSP70, HSP90, cathepsin L1, cathepsin D, casein kinase, and calmodulin were significantly higher in the oncosphere than in the adult or protoscolex (*p* < 0.05). We hope our findings will help to identify potential targets for diagnosis or for therapeutic and prophylactic intervention.

## Abstract (fr)

L’échinococcose kystique, causée par une infection au stade larvaire du cestode *Echinococcus granulosus*, est une zoonose chronique. Le cycle de vie du parasite *E. granulosus* comprend trois étapes consécutives qui nécessitent une régulation génétique ou une expression de protéines spécifiques pour survivre aux changements environnementaux entre les hôtes définitifs et les hôtes intermédiaires. Le but de la présente étude est de cribler et d’analyser les antigènes différentiels de stade à considérer pour le développement de vaccins contre *E. granulosus*. En utilisant la méthode iTRAQ (étiquettes isobares pour la quantification relative et absolue), les protéines différentiellement exprimées ont été sélectionnées parmi les trois stades de développement consécutifs d’*E. granulosus* : l’oncosphère, les ténias adultes et le protoscolex. Grâce à une analyse bioinformatique comprenant les grappes de groupes orthologues (COG), l’ontologie des gènes (GO) et l’annotation des voies métaboliques, nous avons identifié certaines protéines d’intérêt à chaque étape. Les résultats ont montré qu’un grand nombre de protéines exprimées différentiellement (375 : oncosphère vs adulte, 346 : oncosphère vs protoscolex et 391 : adulte vs protoscolex) sont identifiées pour les trois étapes principales du cycle de vie. L’analyse des voies différentielles des protéines a montré que ces protéines différentielles sont principalement enrichies dans les voies métaboliques, la maladie de Huntington, la maladie d’Alzheimer et les voies métaboliques des ribosomes. Fait intéressant, parmi ces protéines différentielles, les niveaux d’expression des protéines paramyosine, HSP60, HSP70, HSP90, cathepsine L1, cathepsine D, caséine kinase et calmoduline étaient significativement plus élevés dans l’oncosphère que chez l’adulte ou le protoscolex (*p* < 0,05). Nous espérons que nos résultats pourront identifier des cibles potentielles pour un diagnostic ou une intervention thérapeutique ou prophylactique.

## Introduction

Cystic echinococcosis (CE) is a severe parasitic zoonosis caused by infection with the larval stage of *Echinococcus granulosus sensu lato* [55], resulting in the development of CE cysts in domestic animals and humans [27]. The life cycle of *E. granulosus* is complex and involves two hosts: definitive and intermediate. Infection with *E. granulosus* occurs after oral ingestion of infective eggs (an oncosphere containing invasive hexacanths [11]). The parasite then hatches in the small intestine of the intermediate host, penetrates the mucosal tissue, enters the blood circulation system, and then migrates to various organs (e.g. liver, lungs, etc.), where it develop cysts filled with fluid and protoscoleces. However, in definitive hosts like dogs, adult parasites develop from the protoscolex in the intestine [7, 41]. Oncosphere, adult and protoscolex are, therefore, the three consecutive stages in the life history of *E. granulosus*.

*Echinococcus granulosus* infection of the host is a complex dynamic process, which involves the recognition and interaction of a variety of biological molecules, including genes [32], and miRNAs [33, 38], which are of great significance for understanding the interaction mechanism between the parasite and the host, and for identifying the key factors of immune regulation and immune evasion, since up-regulation or down-regulation of these molecular expressions likely underpin the phenotype changes associated with the different life cycle stages [3, 63]. If the antigens and candidate vaccine markers specifically expressed in each developmental stage of *E. granulosus* can be screened from the perspective of molecular biology, this could provide the theoretical and practical basis for early diagnosis, preparation of new vaccines, and disease-resistant breeding of livestock.

Recently, the publication of the genome and transcriptome of *E. granulosus* has offered meaningful insights into the development [42, 50], immunobiology, evolution, and mechanisms of host-parasite crosstalk, which provided new information on the development of new, effective treatments and interventions for echinococcosis control. However, there may still be deficiencies in currently available genomic resources for *E. granulosus* [20]. After this landmark step, a series of studies including on miRNA expression [12, 38] and transcriptome analysis [17] were subsequently published for different developmental stages of *E. granulosus.* However, very few proteomic studies have been published on *E. granulosus*, although numerous studies on proteomic profile analysis have been carried out on a wide range of parasites, such as *Mesocestoides* [30, 52]*, Hymenolepis diminuta* [47, 48]*, Schistosoma japonicum* [31], *Trichuris muris* [16], *Toxoplasma gondii* [19, 51], and *Taenia solium* [46]. Protein-compositions have been reported and some of the proteins are considered novel candidate diagnostic antigens [8, 35], or valuable tools for the identification of new therapeutic targets for the control of larval parasites [5, 53]. It is, therefore, necessary to detect the protein expression of *E. granulosus* at the different developmental stages, which may provide information to better understand host-parasite interrelationships, and may point out potential targets for vaccines or drug discovery studies.

In the present study, we aim to characterize the differential expression proteins involved in the development of *E. granulosus*. We provide the iTRAQ-based proteomic analysis of proteins present in the three consecutive stages of *E. granulosus*: oncosphere, adult, and protoscolex. This work provides important information on *E. granulosus* developmental biology, and also contributes to new strategies and targets to combat *E. granulosus* infections in humans and animals.

## Materials and methods

### Ethics statement

This study was performed in strict accordance with the recommendations of the National Institutes of Health Guide for the Care and Use of Laboratory Animals (NIH Publications No. 8023, revised 1978), and the Animal Care and Use Committee of Shihezi University approved all procedures and experiments (Approval number A2018-138-1). Local strain dogs were purchased from farmers around Shihezi city. The owners of the dogs provided oral consent for the use of their dogs in this research, after the purpose of the research was explained to them, and after they were reassured concerning the welfare of animals.

### Preparation and purification of *E. granulosus* at different developmental stages

*Echinococcus granulosus sensu stricto* was maintained through a dog-sheep life cycle in our animal house. Local strain dogs were purchased from farmers around Shihezi city. Before the study, the dogs were confirmed not to be infected with *E. granulosus* by ELISA. Each dog was orally administered 200 mL CE cyst fluid extracted from the fresh liver CE cysts of naturally infected sheep in a slaughterhouse in Shihezi. After 45 days of infection, feces were collected and smeared on a glass slide to observe the ovulation cycle of the eggs.

Preparation of oncosphere and adult samples: on the day before the ovulation of the worm, half the infected dogs were anesthetized, and the duodenum was surgically extracted and cut into pieces of 3 cm under sterile conditions. After bathing in normal saline at 37 °C for 4 h, the adult worms were moved from the duodenum tissue to normal saline. They were aspirated with a plastic-tip dropper, and prepared for microscopic examination under a microscope at 40× and 100×. If a large number of mature gravid proglottid and eggs were observed, the sample was crushed, and saline and isotonic Percoll solution were added; after centrifugation at 3000 r/min, the eggs were collected. Oncosphere activation and cultivation were conducted according to the report published by Heath and Lawrence [23].

Similarly, on the day after worm ovulation, adults without gravid proglottid and eggs were prepared as adult samples. The adult and oncosphere samples were frozen in liquid nitrogen.

Preparation of protoscolex samples: fertile CE cyst were obtained from the livers of naturally infected sheep. The cyst fluid containing protoscoleces was aspirated using a syringe and transferred to a 50-mL centrifuge tube, and centrifuged at 3000 r/min for 5 min; the supernatant was then discarded and the pellet was resuspended in sterile normal saline and centrifuged. The operation was repeated 3 times, and the precipitate was filtered through a 200-μm pore size filter. After the protoscoleces were prepared for microscopic examination under a microscope at 40× and 100×, a sample of protoscolex was cryopreserved in liquid nitrogen.

### Protein extraction

The parasite stages were lysed in lysis buffer (150 mM NaCl, 1.0% Nonidet P-40, 50 mM Tris-Cl, 1% Trasyllol, adjust pH to 7.4), dialyzed, desalted with a Strata X C18 column (Phenomenex, Torrance, CA, USA), and digested with trypsin. Strong cation-exchange (SCX) liquid chromatography fractionation of tryptic peptides was performed.

### iTRAQ labeling and SCX fractionation

Total protein (100 μg, determined by Bradford protein assay) was extracted from each sample solution, and the protein then digested with Trypsin Gold (Promega, Madison, WI, USA) at 37 °C for 16 h. After trypsin digestion, peptides were dried by vacuum centrifugation. Peptides were reconstituted in 0.5M TEAB and processed according to the manufacturer’s protocol for 8-plex iTRAQ reagent (isobaric tags for relative and absolute quantification). Briefly, one unit of iTRAQ reagent was thawed and reconstituted in 24 μL isopropanol. Samples were labeled with the iTRAQ tags as follow: oncosphere, adult and the protoscolex. The peptides were labeled with the isobaric tags, and incubated at room temperature for 2 h. The labeled peptide mixtures were then pooled and dried by vacuum centrifugation. SCX chromatography was performed with an LC-20AB HPLC Pump system (Shimadzu, Kyoto, Japan), according to the manufacturer’s instructions.

### Liquid chromatography-electrospray ionization–tandem mass spectrometry (LC-ESI-MS/MS) analysis

Fractions obtained from the strong cation-exchange column were pooled, lyophilized, and reconstituted in 20 μL 0.1% TFA before analysis by nanobore HPLC-MS/MS, using an LC-20AD nano HPLC (Shimadzu, Kyoto, Japan), an Agilent 1100 Nanoflow LC system coupled online with a linear ion trap–Fourier transform mass spectrometer. Data acquisition was performed with a TripleTOF 5600 System (AB SCIEX, Concord, ON, Canada) fitted with a Nanospray III source (AB SCIEX) and a pulled quartz tip as the emitter (New Objectives, Woburn, MA, USA).

### Data analysis and quantification of proteins

Raw data files acquired from the Orbitrap were converted into MGF files using Proteome Discoverer 1.2 (PD 1.2, Thermo Fisher Scientific Inc., Waltham, MA, USA). Protein identification was performed using the Mascot search engine (Matrix Science, London, UK; version 2.3.02) against the Uniprot Echinococcus database (http://www.uniprot.org/taxonomy/?query=echinococcus&sort=score).

The quantitative protein ratios were weighted and normalized by the median ratio in Mascot. Ratios with *p*-value < 0.05, and only fold changes of >1.2 were considered significant.

Functional annotations of the proteins were conducted using the Blast2GO program against the non-redundant protein database (NR; NCBI). The KEGG database (http://www.genome.jp/kegg/) and the COG database (http://www.ncbi.nlm.nih.gov/COG/) were used to classify and group these identified proteins.

## Results

### Summary of peptide data and protein characterization

**Figure 1:**
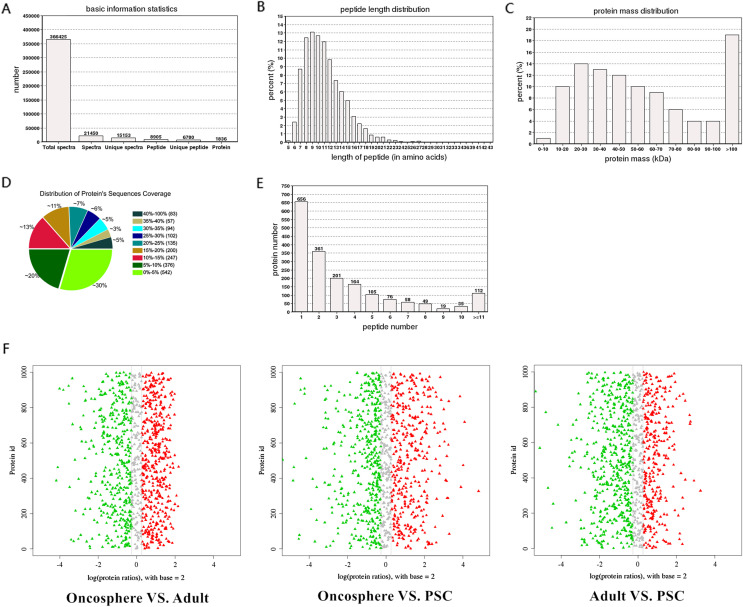
Basic information of protein characterization in *Echinococcus granulosus*. **A**. The identification of basic information of peptides of *Echinococcus granulosus*. The *x*-axis is the identification category, and the *y*-axis the quantity. Total spectra is the total number of secondary spectra, spectra is the number of matched spectra, unique spectra is the number of spectra matched to a unique peptide, peptide is the number of peptides identified, and unique peptide is the number of unique peptide sequences identified. Protein is the number of proteins identified. **B**. Peptide length distribution of *Echinococcus granulosus*. The *x*-axis is the number of amino acid residues in the peptide, and the *y*-axis the percentage of peptides of this length in all peptides. **C**. Proteins mass distribution of *Echinococcus granulosus*. The *x*-axis is the molecular mass of the identified protein (unit: kilodalton, kDa), and the *y*-axis the number of the identified protein. **D**. Distribution of protein sequence coverage of *Echinococcus granulosus*. Different colors represent different protein sequence coverage ranges, and the percentage of the pie chart shows the ratio of the number of proteins in different coverage ranges to the total number of proteins. **E**. Peptide number distribution of *Echinococcus granulosus*. The *x*-axis is the range of the number of peptides identified in the protein, and the *y*-axis is the number of proteins. **F**. Abundance of differentially expressed proteins between groups*.* The *x*-axis represents the value of the difference multiple after logarithm conversion with 2 as the base. Values greater than 0 are up-regulated, and those less than 0 are down-regulated. Among them, points with a multiple of difference greater than 1.2 are marked with red and green (red is increase in expression, and green is decrease in expression).

**Figure 2:**
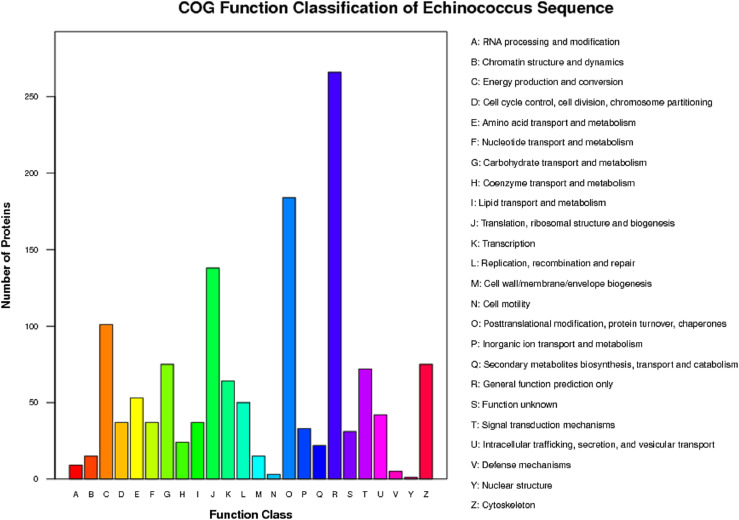
COG Analysis results of identified proteins in all sample groups. The *x*-axis is the COG classification item, and the *y*-axis the protein quantity corresponding to the functional classification. The graph shows the statistical number of proteins with different functions in the sample.

In the present study, using the iTRAQ method, the differentially expressed proteins were selected from the three consecutive stages: oncosphere, adult, and protoscolex of *E. granulosus.* Basic protein information in each group is presented in Figures 1A–1F. The identified proteins were subjected to Gene Ontology (GO), COG analysis, and pathway annotation. The results showed that in the molecular function ontology of proteins, the number of proteins (353) enriched in cellular process entries was the largest, accounting for 18.70% of the total, followed by metabolic process and single-organism process, respectively enriched 310 and 193 proteins, each accounting for 16.42% and 10.23%. COG analysis of identified proteins in all sample groups is shown in Figure 2.

### Differential expression proteins at different developmental stages

We then carried out stages comparison to detect the specifically stage expressed proteins. Based on the screening condition mentioned above, the number of differentially expressed proteins identified between different stages is listed in Table 1.

**Table 1:** Differentially expressed protein analysis between different developmental stages of *E. granulosus*.

Comparison sample groups	Up	Down	Total number of differentially expressed proteins
Oncosphere vs. Adult	213	162	375
Oncosphere vs. protoscolex	167	179	346
Adult vs. protoscolex	127	264	391

Oncosphere vs. adult: a total of 375 differentially expressed proteins were observed between oncosphere and adult *E. granulosus* (without eggs), with 213 up-regulated and 162 down-regulated (see Data Analysis, ratios with *p*-value < 0.05, and only fold changes of >1.2 were considered significant differences). The partial significant differential expression proteins are listed in Table 2, and the partial up-regulated proteins were diagnostic antigen gp50, dynein light chain, and thioredoxin glutathione reductase. Metabolic pathways showed that 285 differential proteins were assigned in 188 pathways, with 81 in metabolic pathways, 66 in three diseases, and 21 in ribosome (Table 3). Following GO analysis, partial representative GO terms of differentially expressed proteins were assigned as “structural molecule activity” and “oxidoreductase activity” (Table 6).

**Table 2:** Partial significant differential expression proteins between different developmental stages of *E. granulosus.*

Comparison sample groups	Up or Down	Proteins	Multiple of difference	Score
Oncosphere vs. Adult	Up	Diagnostic antigen gp50	4.53	73
	Up	Dynein light chain	4.436	125
	Up	Thioredoxin glutathione reductase	1.709	176
	Down	Clathrin	0.798	590
	Down	RNA-binding protein	0.781	58
	Down	Calcium-transporting ATPase	0.77	758
Oncosphere vs. protoscolex	Up	Putative HSP20 related protein	8.582	717
	Up	Actin-binding and Severin family Group-like protein	2.99	725
	Up	Serine protease inhibitor	2.664	271
	Down	40S ribosomal protein S9	0.83	142
	Down	Receptor mediated endocytosis family member	0.794	90
	Down	Glutathione S-transferase	0.563	296
Adult vs. protoscolex	Up	Cysticercus cellulosae specific antigen	6.62	220
	Up	Coiled-coil domain-containing protein 61	6.43	22
	Up	Major egg antigen p40	5.513	717
	Down	Aldehyde dehydrogenase, mitochondrial	0.756	363
	Down	KH domain-containing RNA binding signal	0.754	65
	Down	Ferritin	0.357	541

**Table 3:** Major pathway analysis of differentially expressed proteins between oncosphere and adult of *E. granulosus*.

Comparison sample groups	Pathways	Differential protein quantity and percentage of total	*p*-value	Pathway ID
Oncosphere vs. Adult	Metabolic pathways	81 (28.42%)	0.000537	ko01100
	Parkinson’s disease	22 (7.72%)	0.004300595	ko05012
	Alzheimer’s disease	22 (7.72%)	0.0237847	ko05010
	Huntington’s disease	22 (7.72%)	0.03240847	ko05016
	Ribosome	21 (7.37%)	0.03289107	ko03010

**Table 4:** Major pathway analysis of differentially expressed proteins between oncosphere and PSC of *E. granulosus*.

Comparison sample groups	Pathways	Differential protein quantity and percentage of total	*p*-value	Pathway ID
Oncosphere vs. protoscolex	Metabolic pathways	81 (30%)	6.48E-05	ko01100
	Dilated cardiomyopathy	19 (7.04%)	5.43E-06	ko05414
	Focal adhesion	19 (7.04%)	0.141204	ko04510
	Hypertrophic cardiomyopathy (HCM)	18 (6.67%)	2.60E-05	ko05410
	Phagosome	18 (6.67%)	0.002149776	ko04145

**Table 5:** Major pathway analysis of differentially expressed proteins between adult and protoscolex of *E. granulosus*.

Comparison sample groups	Pathways	Differential protein quantity and percentage of total	*p*-value	Pathway ID
Adult vs. protoscolex	Metabolic pathways	82 (29.29%)	0.000154	ko01100
	Parkinson’s disease	20 (7.14%)	0.016668	ko05012
	Huntington’s disease	20 (7.14%)	0.084439	ko05016
	Alzheimer’s disease	18 (6.43%)	0.174785	ko05010
	Focal adhesion	18 (6.43%)	0.266034	ko04510

**Table 6:** Partial representative GO terms of differentially expressed proteins between oncosphere and adult of *E. granulosus*.

Annotations number	Gene ontology term	*p*-value	Accession
GO:0005198	Structural molecule activity	0.004745	hCG1990955, isoform CRA_a
			Tubulin beta
			Tubulin beta-2
			Tubulin alpha-1C chain-like isoform 2
			Tubulin alpha-1D chain
			Tubulin alpha-1C chain-like
			alpha-Tubulin
			Putative ribosomal protein L30
			40S ribosomal protein S4-like protein
			Ribosomal protein S3
			60S ribosomal protein L21
			40S ribosomal protein S6
			60S ribosomal protein l11
			Ribosomal protein S5a
			60S ribosomal protein L18
			Ribosomal protein L3
			Hypothetical protein
			Large subunit ribosomal protein 23
			60S ribosomal protein l31
			Mitochondrial H+ transporting ATP synthase F1 complex alpha subunit 1
			Chaperonin GroEL
			Ornithine aminotransferase, partial
			T-complex protein 1 subunit gamma, partial
			Molecular chaperone DnaK
			T-complex protein 1 subunit alpha
			Elongation factor 2
			Adenylate kinase
			Aspartate aminotransferase mitochondrial
			Chaperonin containing t-complex protein 1 epsilon subunit tcpe
			DEAD box ATP-dependent RNA helicase
			Chain A, Gdp-Bound Rab14 Gtpase
			Similar to Drosophila melanogaster Cam, partial
			Ras-related nuclear protein
			Pgd protein
			DEAD box RNA helicase Me31B
			Nucleoside-diphosphate kinase
			Translation initiation factor 2 subunit 3
			Cdc42-like protein
			Arl1 protein
			Putative ADP-ribosylation factor 6 variant 3
			Spliceosome RNA helicase bat1
			cAMP-dependent protein kinase catalytic subunit
			GF14408
			Glyceraldehyde-3-phosphate dehydrogenase
			Lactate dehydrogenase A
			Malate dehydrogenase
			Pyruvate dehydrogenase
			Putative malate dehydrogenase, partial
			Lactate dehydrogenase B
			ATP:guanidino kinase (Smc74)
			Gyceraldehyde-3-phosphate dehydrogenase
			Succinate dehydrogenase iron-sulfur protein
			Thioredoxin glutathione reductase
			Peroxiredoxin

Oncosphere vs. protoscolex: 346 differentially expressed proteins were characterized, of which 167 are up-regulated and 179 are down-regulated. Metabolic pathways showed that proteins are involved in pathways including metabolic, dilated cardiomyopathy, focal adhesion, hypertrophic cardiomyopathy (HCM), and phagosome (Table 4). Table 2 represents partial significant differential expression proteins, which were putative HSP20 related protein, actin-binding and Severin family group-like protein, and serine protease inhibitor. Following GO analysis, partial representative GO terms of differentially expressed proteins were assigned as “oxidoreductase activity” and “structural molecule activity” (Table 7).

**Table 7:** Partial representative GO terms of differentially expressed proteins between oncosphere and protoscolex of *E. granulosus*.

Annotations number	Gene ontology term	*p*-value	Accession
GO:0016491	Oxidoreductase activity	0.096062	Pgd protein
			Lactate dehydrogenase A
			Malate dehydrogenase
			Putative malate dehydrogenase, partial
			Lactate dehydrogenase B
			Hypothetical protein AND_22292
			ATP:guanidino kinase (Smc74)
			Gyceraldehyde-3-phosphate dehydrogenase
			Succinate dehydrogenase iron-sulfur protein
			Peroxiredoxin
			Isocitrate dehydrogenase
			Glutamate dehydrogenase
			NADH-ubiquinone oxidoreductase 75 kDa subunit, mitochondrial-like, partial
			Malic enzyme
			Putative isocitric dehydrogenase subunit alpha
GO:0005198	Structural molecule activity	0.006014	Tubulin beta
			Tubulin alpha-1D chain
			Tubulin alpha-1C chain-like
			alpha-Tubulin
			40S ribosomal protein S4-like protein
			60S ribosomal protein L21
			40S ribosomal protein S6
			Ribosomal protein S5a
			Hypothetical protein CAPTEDRAFT_163836, partial
			Tubulin beta-3 chain
			EG10
			Myosin-like protein
			Ribosomal protein S8
			Pyrroline-5-carboxylate reductase
			Ribosomal protein S9
			Small subunit ribosomal protein S16e
			Chaperonin GroEL
			T-complex protein 1 subunit alpha
			Adenylate kinase
			Aspartate aminotransferase mitochondrial
			DEAD box RNA helicase Me31B
			Spliceosome RNA helicase bat1
			cAMP-dependent protein kinase catalytic subunit
			GF14408
			Enolase
			Starch phosphorylase, partial
			Elongation factor 1 alpha
			ADP-ribosylation factor-like
			Hypothetical protein PANDA_003774
			Cytidine deaminase
			Trimeric G-protein alpha o subunit
			Guanine nucleotide binding protein (G protein) alpha o polypeptide
			Hypothetical protein PANDA_008713
			Carboxypeptidase A1
			Calreticulin, partial
			Cytochrome c
			Centractin alpha, partial
			GTP binding protein

Adult vs. protoscolex: a total of 391 differentially expressed proteins were observed between adults and the protoscolex, with 127 up-regulated and 264 down-regulated. Metabolic pathways showed that they are involved in metabolic, several diseases, and focal adhesion pathways (Table 5). Partial significant differential expression proteins are listed in Table 2. Following GO analysis, partial representative GO terms of differentially expressed proteins were assigned as “oxidoreductase activity (including acting on the CH-OH group of donors, NAD or NADP as acceptor”, and “ion binding” (Table 8).

**Table 8:** Partial representative GO terms of differentially expressed proteins between Adult and protoscolex of *E. granulosus*.

Annotations number	Gene Ontology term	*p*-value	Accession
GO:0016616	Oxidoreductase activity, acting on the CH-OH group of donors, NAD or NADP as acceptor	0.000446	Pgd protein
			Isocitrate dehydrogenase
			Malate dehydrogenase
			Putative malate dehydrogenase, partial
			Lactate dehydrogenase B
			Malic enzyme
			Malate dehydrogenase (oxaloacetate-decarboxylating)(NADP+), partial
GO:0043167	ion binding	0.009184	hCG1990955, isoform CRA_a
			Tubulin alpha-1D chain
			Tubulin alpha-1C chain-like
			alpha-Tubulin
			Tubulin beta-3 chain
			Putative isocitric dehydrogenase subunit alpha
			Aspartate aminotransferase mitochondrial
			DEAD box RNA helicase Me31B
			Spliceosome RNA helicase bat1
			Enolase
			Starch phosphorylase, partial
			ADP-ribosylation factor-like
			Hypothetical protein PANDA_003774
			Cytidine deaminase
			Carboxypeptidase A1
			Cytochrome c
			Centractin alpha, partial
			Tubulin beta-2 chain
			Tubulin alpha-1C chain-like isoform 2
			T-complex protein 1 subunit gamma, partial
			Molecular chaperone DnaK
			Chaperonin containing t-complex protein 1 epsilon subunit tcpe
			Chain A, Gdp-Bound Rab14 Gtpase
			Similar to Drosophila melanogaster Cam, partial
			Arl1 protein
			Putative ADP-ribosylation factor 6 variant 3
			Myosin regulatory light chain
			Myosin essential light chain striated adductor muscle
			T-complex protein 1 subunit zeta
			Ras-related protein Rab-7a
			Hypothetical protein
			GE25707
			SAR1 gene homolog B (S. cerevisiae), isoform CRA_a
			V-type ATPase A subunit isoform 2
			Ras-related C3 botulinum toxin substrate 2
			Ras-related protein Rab-6A, partial
			Translation initiation factor 4A-like protein

Interestingly, analysis of the differential protein pathways showed that these differential proteins are mainly enriched in metabolic pathways, Huntington’s diseases, Alzheimer’s diseases, and ribosome metabolic pathways. Among these differential proteins, the expression levels (determined by protein quantifications, details shown in section “Materials and methods”) of paramyosin, HSP60, HSP70, HSP90, cathepsin L1, cathepsin D, casein kinase and calmodulin were significantly more highly expressed at specific development stages and attracted our attention.

## Discussion

*Echinococcus granulosus* is an important parasite that causes cystic echinococcosis in domestic animals and humans. Although the research on genomic analysis of *E. granulosus* has provided significant insights into the occurrence and development, immunobiology [50], evolution and mechanisms, there are also reports that there are still outstanding deficiencies in the available genomic resources [20]. Similar to other reports [26], identification and characterization of the proteins expressed by the main stages of *E. granulosus* might help to understand the complexity of the parasites and their interactions with the host [36]. It is also helpful to identifying new candidates for immunodiagnosis and vaccine development.

Paramyosin, also known as tropomyosin A, is the muscle constituent protein of invertebrates such as molluscs and annelids [9]. Studies have shown that paramyosin is expressed in the epidermis, subcutaneous tissues and organs of many parasites, and can also be secreted *in vitro* [60]. Pearce et al. [44] obtained paramyosin from soluble adult worm antigen of *Schistosoma mansoni* by affinity chromatography, and preliminarily elucidated the cellular immune mechanism of this protein. Nanduri and Kazura [40] found that the protein can enhance the ability of mice to remove the microfilaria of *Brugia malayi*. It has been demonstrated that paramyosin is distributed throughout the base layer in the oncosphere and adult of *E. granulosus* [39], and it can also be used as an important antigen for parasites to trigger the host immune response [62]. Paramyosin can cause a strong immune response, and has been selected as a parasite candidate antigen [28, 49]. However, studies on paramyosin in *E. granulosus* have rarely been reported.

In our study, we found that paramyosin were significantly expressed in the oncosphere, when compared with adults and the protoscolex, respectively. The expression level of the protein between the oncosphere and adults differed by 2.906 times, while between the oncosphere and the protoscolex there was a difference of 3.224 times, reaching a significant level of difference (*p* < 0.05). A previous report showed that the paramyosin of *Trichinella spiralis* can evade immune attack by combining with the host complement during the process of infecting mice [64], similarly as in *Schistosoma japonicum* [45]. We speculated that it appears in the oncosphere with strong invasion ability. The highly expressed protein may also have similar effects. In addition, the host infected by *Echinococcosis* has an obvious feature, i.e., after the formation of stable solid endoparasites (adults) and intractable lesions (CE cyst) at the later stage of infection, the host’s immune attack and rejection capabilities are significantly reduced. We speculated that the paramyosin protein antibodies may become candidate markers for detecting echinococcosis infection during the early stage of infection, especially for the oncosphere. Meanwhile, there is also potential for paramyosin to become an epitope vaccine against CE, as other reports have shown in other parasites and hosts [21, 56].

Heat shock proteins are a class of heat emergency proteins widely found in parasites and mammals. When an organism is exposed to high temperatures, it will be stimulated by heat to synthesize this protein to protect itself. Studies have shown that many heat shock proteins could participate in the process of worm differentiation and can enhance its virulence, and can also be used as main protein antigens [29, 57, 58]. Among them, HSP70 is especially focused in parasite diseases such as *Schistosoma japonicum* infection [18], and it has a positive regulatory effect on the immune evasion of parasite larvae [42, 47].

In the present study, we found that the expression levels of HSP60 and HSP70 proteins in the samples of the oncosphere were significantly higher than that of the samples of adults and the protoscolex of *E. granulosus,* reaching a significant level of difference (*p* < 0.05). We speculate that the reason for this difference in expression may be due to the fact that, under natural conditions, the eggs of the *E. granulosus* enter the digestive tract of the body after being swallowed by the host from the external environment, and they are stimulated by digestive juices to shed their shells and hatch into oncospheres. In this process, with the loss of the eggshell protection, the change of environmental temperature and physicochemical conditions, as well as the killing effect of the host immune response, put the oncosphere under stress, which leads to the high expression of HSP60 and HSP70 proteins to enhance virulence and immune evasion. Subsequently, the oncosphere develops into recalcitrant parasites such as the adult and protoscolex in intermediate and definitive hosts; the living environment is relatively stable, the stress state gradually subsides, and the expression levels of the two proteins decrease. As a result of this change in expression, it is speculated that HSP60 and HSP70 can be used as a prognostic indicator to detect the early stage infections of intermediate and definitive hosts [43], and could also be considered a vaccine candidate for protection [25].

In addition, we found that the expression level of HSP90 protein presented no difference between the samples of the oncosphere and adults, while the expression was higher in adults than the protoscolex, reaching a significant level of difference (*p* < 0.05). Since adults parasitize primarily in the intestinal tissues of intermediate hosts, and protoscoleces parasitize primarily in liver tissues of definitive hosts, it is speculated that the differential expression of HSP90 may be related to the tissue specificity of parasitism.

Cathepsin is a type of protease found in the cells of various animal tissues (especially the lysosomal part). Studies have showed that cathepsin participates in many special physiological processes, such as the activation of prohormones, antigen presentation, and immunity of many diseases such as tumor infiltration and metastasis, and parasitic infections [22, 34].

In this study, we found that the expression levels of three cathepsin subtypes L1, D, and B in the oncosphere were higher than those in adults. In consideration of the structural and functional characteristics of the three cathepsins, we inferred that during the host infection process of the oncosphere, cathepsin L1 is released into the cytoplasmic or interstitial spaces, degrading cellular components or intercellular matrix components, so that the oncosphere can pass intestinal epithelial cells and enter the capillaries to reach the blood circulatory system. Cathepsin D can not only use hydrolysis to degrade the extracellular matrix, but also activate cathepsin B. Cathepsin B can act on major histocompatibility complex II (MHC-II) related constant chains to produce an effect on antigen processing and delivery. The presentation process is blocked, which may reduce the level of host immune response to protect the parasite.

Casein kinase (CK) is a conserved silk/threonine protein kinase of eukaryotes, which is involved in the regulation of a variety of important cellular processes [6], including the wnt (Wingless-Type MMTV integration site family) signaling pathway, membrane transport, and cytoskeleton maintenance [10]. Recent studies have found that CK1 gene expression is related to parasite infections, like *Toxoplasma gondii* [15], *Plasmodium* [24], *Leishmania* [1], and *Trypanosoma* [14]. Highly expressed CK1 inhibitors can inhibit the proliferation of flagellates and trypanosomes in the blood of the host. Studies have shown that the high expression of CK has a positive regulatory effect on the immune escape of parasites [61].

We found that the expression level of casein kinase 1 (CK1) in the oncosphere was higher than that of adults by 2.435 times. In addition to the immunosuppressive effect of CK1 in the body of the oncosphere, combined with its structural and functional characteristics, we speculate that CK1 can also promote the adhesion of oncospheres to the surface of small intestine cells and tissue invasion, which is similar to the findings of a study on *Toxoplasma gondii* reported by Wang et al. [61].

Calmodulin is a protein that can bind to calcium ions, the second messenger in the cell [37]. It has been reported that calmodulin is related to the motility of parasites during the development of parasites and plays an important role in the formation of eggs [2, 4].

In our study, we found that calmodulin did not express differentially between the oncosphere and adults, but the expression level in adults was higher than that in the protoscolex with a fold of 3.779. In consideration of its structure and function, we speculate that the high expression of this protein can promote the motility of the oncosphere so as to cross the intestinal mucosa faster and enhance its infiltration ability. During the development of adults, it promoted the maturation of the pregnant section and the formation of eggs. The mature protoscolex mainly exists in the cysts of the host’s liver and other organs, and the parasitic environment is relatively stable, which may cause the down-regulation of calmodulin expression. This finding is similar to that in a study on the function of calmodulin in *Clonorchis sinensis* reported by Zhou et al. [65].

Taken together, in our study, by using iTRAQ technology, proteins such as paramyosin, heat shock proteins, and cathepsin were specifically expressed in the oncosphere, which may play a role in adaption or evasion from host attack in the early stages of infection, and these are likely vaccine candidates for further study.

Although technologies like LC-MS/MS and 2-DE-gel have been used to study the protein composition of *E. granulosus* at a certain developmental stage [9, 54, 59], and extended the existing proteomic data [13], there are few reports on the regular pattern of the screened proteins during the consecutive development of the parasites. This study could help in understanding how *E. granulosus* adapts to its different and complex parasitic life cycles, and universal molecules among diverse groups of cestodes to escape from host immune response.

The iTRAQ method has obvious advantages such as high throughput, strong separation ability, and wide analysis range, which improves the reliability of the data. In our research, using this technology to analyze samples can obtain more target peptides than conventional traditional methods, as 6780 unique peptide segments and 1836 protein sequences were identified from three consecutive developmental stages of *E. granulosus*, and many differentially expressed proteins were screened. Further analysis showed that these differential proteins were enriched into multiple metabolic pathways related to target site recognition, disease occurrence, and immune response. The results may provide a rich resource to underpin the development of urgently needed treatments to control parasite infection.

## Conflict of interest

The authors declare that they have no conflicts of interest to this work: we declare that we do not have any commercial or associative interest that represents a conflict of interest in connection with the work submitted.
